# Final Residues, Storage Stability, and Dietary Exposure of Pyroxasulfone and Diflufenican in Wheat Grain and Straw

**DOI:** 10.3390/foods15040732

**Published:** 2026-02-16

**Authors:** Min He, Ping Han, Li Chen, Pingzhong Yu, Junxue Wu, Fajun Tian, Xiaotong Qin, Ercheng Zhao

**Affiliations:** 1 Institute of Plant Protection, Beijing Academy of Agriculture and Forestry Sciences, Beijing 100097, China; hanping@baafs.net.cn (P.H.); chenli@baafs.net.cn (L.C.); yupingzhong@baafs.net.cn (P.Y.); wujunxue@baafs.net.cn (J.W.); qinxiaotong0622@163.com (X.Q.); zhaoercheng@baafs.net.cn (E.Z.); 2 Zhengzhou Fruit Research Institute, Chinese Academy of Agricultural Sciences, Zhengzhou 450009, China; 3 College of Plant Health and Medicine, Qingdao Agricultural University, Qingdao 266109, China

**Keywords:** pesticide residue analysis, metabolites, nationwide field trials, storage stability, dietary risk assessment

## Abstract

This study developed a UHPLC-MS/MS method combined with a modified QuEChERS pretreatment technique to simultaneously determine diflufenican, pyroxasulfone, and their metabolites (M-1, M-3, M-25) in wheat grain and straw. The method exhibited good linearity within the concentration range of 0.0001–0.1 mg/L (*r* > 0.996), with a limit of quantification of 0.002 mg/kg. At three spiked levels, the average recoveries of the target analytes ranged from 82.0% to 110.1%, with relative standard deviations ≤ 13.9%. All analytes remained stable in both grain and straw matrices when stored at ≤−18 °C for 12 months. Field trials demonstrated that diflufenican residues in grains were all below 0.002 mg/kg, while residues in straw ranged from 0.002 to 0.018 mg/kg. The total residues of pyroxasulfone and its metabolites in grains ranged from 0.010 to 0.019 mg/kg, and in straw from 0.026 to 0.357 mg/kg. Dietary risk assessment results showed that the risk quotients for diflufenican and pyroxasulfone were 0.10% and 0.27%, respectively, both far below the 100% safety threshold. Based on the residue data and risk assessment results, it is recommended that the maximum residue limits for pyroxasulfone in wheat in China be set at 0.03 mg/kg for grains and 0.6 mg/kg for straw. These proposed values align with international standards while also accommodating the practical needs of domestic agricultural production and regulatory oversight.

## 1. Introduction

Winter wheat is a crucial summer grain crop in China, playing a pivotal role in ensuring national food security. Weeds are a major constraint on the yield and quality of winter wheat, and chemical weed control remains the primary approach in current field management due to its operational simplicity and significant efficacy [1,2]. In recent years, the increasing prominence of herbicide resistance has made the development of new herbicide mixtures and formulations a key strategy for delaying resistance development and enhancing control effectiveness [3,4,5]. In this context, the 200 g/L pyroxasulfone–diflufenican suspension concentrate (SC) developed by Anhui Zhongtu Chemical Co., Ltd. (Hefei, China) has passed field efficacy validation and demonstrates effective control of weeds in winter wheat fields, providing a new technical solution for managing resistant weed populations.

**Figure 1:**
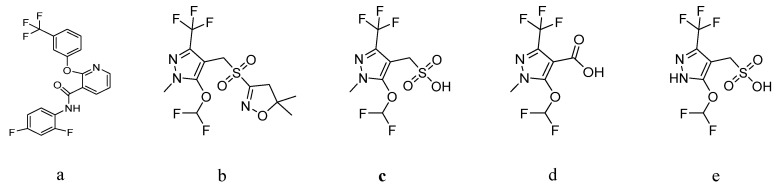
Molecular structures of diflufenican (**a**), pyroxasulfone (**b**), M-1 (**c**), M-3 (**d**), and M-25 (**e**).

Diflufenican (Figure 1a) is a pyridine carboxamide herbicide developed by Bayer CropScience. Its mechanism of action involves the specific inhibition of phytoene desaturase, disrupting carotenoid biosynthesis, which ultimately leads to chlorophyll degradation and plant death [6]. Pyroxasulfone (Figure 1b) is a pre-emergence herbicide belonging to the 3-sulfonylisoxazoline class. It acts as a potent inhibitor of very-long-chain fatty acid elongase, thereby blocking fatty acid synthesis and ultimately compromising cell membrane integrity. Compared to traditional herbicides, pyroxasulfone can effectively control annual grass weeds and certain small-seeded broadleaf weeds at significantly reduced application rates and shows good activity against resistant weed biotypes [7]. In plants, pyroxasulfone metabolizes into characteristic derivatives such as M-1, M-3, and M-25. Among these, M-1 (an isoxazole ring cleavage product, Figure 1c) retains partial herbicidal activity, whereas M-3 (a methyl sulfonyl oxidation product, Figure 1d) and M-25 (a pyrazole ring methyl reduction product, Figure 1e) exhibit significantly reduced activity and demonstrate more environmentally favorable degradation behavior.

Scientific and rational herbicide use is an important technical guarantee for achieving high and stable yields of winter wheat [8,9]. However, the improper application of diflufenican and pyroxasulfone may pose environmental and health risks, primarily manifesting as potential impacts on soil and aquatic ecosystems, as well as direct exposure threats to field workers and non-target organisms [10,11,12]. Long-term, low-dose exposure to herbicides may result in chronic health effects including carcinogenicity, mutagenicity, and endocrine disruption [13,14]. Additionally, the interplay of multiple herbicides and their metabolites in organisms could be synergistic or antagonistic, leading to an amplification of the composite toxicity [15]. To effectively manage pesticide residue risks, maximum residue limits (MRLs) are widely adopted as the core regulatory standard for pesticide residues in food internationally [16,17]. Due to differences in agricultural production methods and dietary structures, MRL standards vary across countries [18]. In establishing pesticide MRLs, China systematically references the regulatory systems of key trading partners, including the Codex Alimentarius Commission, the European Union, the U.S. Environmental Protection Agency, Australia, Japan, and the Republic of Korea [19,20]. This approach has progressively shaped an MRL framework that aligns with international norms while addressing domestic conditions [21].

At present, diflufenican is registered in China for pre-emergence weed control in crops such as wheat, garlic, and rice, while pyroxasulfone is approved for pre-emergence weed management in winter wheat, soybeans, and corn [22]. While China has established MRLs for diflufenican in wheat, no MRLs have yet been set for pyroxasulfone in this crop. MRLs represent the maximum permissible concentration of pesticide residues in agricultural products under Good Agricultural Practices (GAP) and serve as a key scientific and regulatory basis for pesticide registration and market supervision.

Table 1 summarizes and compares the MRLs for diflufenican and pyroxasulfone established by regulatory authorities across different countries and regions. Regarding diflufenican, there is consistent alignment among countries with established standards, as its residue definition refers solely to the parent compound without including metabolites. Among jurisdictions that have set MRLs for pyroxasulfone, Japan and the Republic of Korea adopt a more conservative approach by defining the residue exclusively as the parent compound pyroxasulfone and setting its limit in wheat at 0.01 mg/kg. Australia defines pyroxasulfone residues in wheat as the sum of the compound and its metabolite M-1, with the same MRL of 0.01 mg/kg. In contrast, the regulatory framework established by the U.S. Environmental Protection Agency is more scientifically rigorous and systematic, and its established MRLs are relatively higher: for wheat grain, the residue is defined as the sum of pyroxasulfone and metabolite M-3 (MRL 0.03 mg/kg), while for wheat straw, it includes the sum of pyroxasulfone and its metabolites M-1, M-3, and M-25 (MRL 0.60 mg/kg).

Countries and regions that have established MRLs for diflufenican or pyroxasulfone show significant variations in residue definitions and MRL values. Differences in MRLs and metabolite detection requirements also impose varying technical standards for pesticide residue analysis. Existing analytical methods for determining diflufenican residues, as reported in the literature, employ techniques such as gas chromatography–mass spectrometry (GC-MS) and liquid chromatography–tandem mass spectrometry (LC-MS/MS) across various matrices including wastewater [32], natural water bodies [33], sorghum and rice husk [34], pea [35], wheat [36,37] and soil [38]. For pyroxasulfone, residue analysis in honey is commonly performed using GC-MS/MS [39], whereas HPLC-UV is frequently applied for soil matrices [40]. Nevertheless, most of these methods do not include the determination of key metabolites. Although the EPA has reported an LC-MS/MS method capable of simultaneously determining pyroxasulfone and its primary metabolites M1 and M3 in soil with a limit of quantification (LOQ) of 0.002 mg/kg [41], and Lan et al. used UHPLC-MS/MS to analyze pyroxasulfone and its metabolites in wheat straw and grain [42], the reported LOQ of 0.02 mg/kg is insufficient to meet the sensitivity required for monitoring an MRL of 0.01 mg/kg.

To date, no method has been reported for the simultaneous determination of diflufenican, pyroxasulfone, and their major metabolites (M1, M3, and M25) in wheat straw and grain. Furthermore, studies on the storage stability of these compounds in wheat matrices and their comprehensive dietary risk assessment remain scarce.

Therefore, this study developed and validated a UHPLC-MS/MS method for the simultaneous quantification of pyroxasulfone, diflufenican, and their metabolites (M1, M3, and M25) in wheat straw and grain. The storage stability of these target analytes in wheat matrices under conditions of ≤−18 °C was systematically evaluated. Based on reliable residue data obtained from standardized field trials conducted across ten major winter wheat-producing regions in China during the 2023–2024 growing season, a systematic chronic dietary risk assessment was performed. The results of this study provide scientific guidance for the safe application of 200 g/L pyroxasulfone–diflufenican SC and offer robust data foundation to support regulatory authorities in establishing science-based MRLs for pyroxasulfone, thereby serving as a critical reference for government pesticide management and food safety supervision.

## 2. Materials and Methods

### 2.1. Instruments and Reagents

The 200 g/L pyroxasulfone–diflufenican SC, containing 120 g/L pyroxasulfone and 80 g/L diflufenican, was supplied by Zhongtu Chemical (Anhui) Co., Ltd. (Hefei, China). Analytical standards of diflufenican (CAS No. 83164-33-4, purity 99.9%), pyroxasulfone (CAS No. 447399-55-5, purity 99.7%), and its metabolites M-1 (CAS No. 1379794-40-7, purity 99.9%), M-3 (CAS No. 1379794-41-8, purity 99.0%), and M-25 (CAS No. 1379794-42-9, purity 99.9%), all as 100 mg/L standard solutions in ACN, were purchased from Alta Scientific Co., Ltd. (Tianjin, China). HPLC-grade ACN formic acid (purity ≥ 99.0% for both) were obtained from J&K Scientific Ltd. (Beijing, China). Anhydrous magnesium sulfate (MgSO_4_) and sodium chloride (NaCl), both of analytical grade, were purchased from Sinopharm Chemical Reagent Co., Ltd. (Shanghai, China). A 2 mL purification tube (50 mg C_18_ sorbent) and a 0.22 µm organic syringe filter unit were obtained from Bonna-Agela Technologies Co., Ltd. (Tianjin, China).

The following instruments and equipment were used in this study: a Waters ACQUITY UPLC^TM^ coupled with a Xevo TQ-XS (Waters Corporation, Milford, MA, USA); an BEH C_18_ column (2.1 mm × 100 mm, 1.7 μm; Waters Corporation, USA); variable-volume pipettes (10, 100, and 1000 μL; Eppendorf, Hamburg, Germany); analytical balances (models MS105DU and XP6, Mettler-Toledo GmbH, Greifensee, Switzerland) with readabilities of 0.01 mg and 0.1 mg, respectively; UMV-2 multi-tube vortex mixer was supplied by Beijing You United Technology Co., Ltd. (Beijing, China). TDZ5-WS benchtop low-speed centrifuge and H1650-W benchtop high-speed centrifuge were obtained from Hunan Xiangyi Laboratory Instrument Development Co., Ltd. (Changsha, China). A high-speed multifunctional grinder (Model FLB-800A, Feilibo Industrial Co., Ltd., Shanghai, China) was employed for sample homogenization.

### 2.2. Field Trials and Sample Collection

Nationwide field trials were conducted across the major winter wheat-producing regions in China, in accordance with the *Guidelines on Pesticide Residue Trials* (NY/T 788-2018) [43]. A single pre-emergence soil application of 200 g/L pyroxasulfone–diflufenican SC was applied at a rate of 480 g of active ingredient per hectare (g a.i./ha) during the winter wheat growing. At each of the ten sites, treated and control plots (each with a minimum area of 100 m^2^) were established and subsequently harvested for comparative analysis. The corresponding field trial parameters are detailed in Table 2.

To ensure sample representativeness and homogeneity, wheat grain and straw samples were collected and prepared during the maturity stage. Within each experimental plot, twelve random samples were collected from each site for analysis. The 12 random samples from each site were collected individually.

Grain samples were collected using scissors, with each sample weighing no less than 1.0 kg. After collecting, the grain were air-dried under indoor conditions, manually threshed, and thoroughly homogenized. The homogenized grain was quartered to obtain two parallel subsamples (designated A and B), each with a minimum weight of 200 g, which were then stored in clearly labeled, sealed bags.

Straw samples were collected concurrently from the same plots by harvesting above-ground whole wheat plants at more than 12 randomly distributed points, with each sample weighing at least 1.0 kg. The straw was trimmed into segments not exceeding 1.0 cm in length using scissors, homogenized, and subsequently subjected to quartering. Two parallel subsamples (designated A and B), each with a minimum mass of 100 g, were thereby prepared.

All procedures followed established laboratory sample-handling protocols to ensure sample integrity, homogeneity, and traceability. The prepared samples were stored at −18 °C, and the storage duration from collection to analysis did not exceed 12 months for any sample.

### 2.3. Analytical Procedures

#### 2.3.1. Extraction and Cleanup

A 5.0 g portion of homogenized grain or 2.0 g of straw (weighed accurately to 0.01 g) was placed into a 50 mL polytetrafluoroethylene (PTFE) centrifuge tube. Then, 20 mL of an ACN-water mixture (3:1, *v*/*v*) was added. The tube was shaken on an orbital shaker at 240 rpm for 30 min for extraction, followed by centrifugation at 4000 rpm for 5 min. The supernatant was collected into a 50 mL centrifuge tube. The extraction procedure was repeated with another 20 mL of the ACN-water mixture (3:1, *v*/*v*). The combined supernatants were thoroughly mixed. A 1.0 mL aliquot of the extract was precisely transferred into a 2 mL d-SPE tube containing C_18_ sorbent. The mixture was vortexed for 1 min and then centrifuged at 12,000 rpm for 3 min. The resulting supernatant was passed through a 0.22 µm organic syringe filter. The filtrate (approximately 1.0 mL) was collected into a pre-slit 2.0 mL autosampler vial for subsequent UHPLC-MS/MS analysis.

#### 2.3.2. Instrumental Condition

The separation was performed on a Waters Acquity UPLC^TM^ BEH C_18_ column at 38 °C, equipped with a Waters Acquity UPLC^TM^ column filter unit at the front end. The autosampler temperature was maintained at 15 °C, and the injection volume was set to 3.0 µL. The mobile phase consisted of ACN (phase A) and 0.1% formic acid in water (*v*/*v*, phase B), delivered at a constant flow rate of 0.3 mL/min. The gradient elution program was set as follows: 0–1.0 min, isocratic elution (10% A); 1.0–3.0 min, linear gradient to 100% A; 3.0–4.0 min, maintained at 100% A for column cleaning; 4.0–5.0 min, linear gradient back to the initial condition (10% A); and 5.0–6.0 min, re-equilibration at 5% A. The total time per sample was 6.0 min, with a post-run extension of 1.0 min to ensure system stability.

Detection was performed using an electrospray ionization (ESI) source in positive/negative switching mode. The capillary voltage was set to 3.62 kV, with the source and desolvation temperatures maintained at 150 °C and 410 °C, respectively. The gas system used high-purity nitrogen as the desolvation gas (680 L/h) and cone gas (180 L/h), with high-purity argon introduced into the collision cell at a flow rate of 0.20 mL/min. In multiple reaction monitoring (MRM) mode, two characteristic ion transitions (quantifier and qualifier) were selected for each compound, combined with retention time to achieve triple confirmation. The optimized mass spectrometry parameters for diflufenican, pyroxasulfone, M-1, M-3, and M-25 are listed in Table 3.

### 2.4. Spike-And-Recovery Experiment

The intermediate mixed stock solution at 5.0 mg/L was prepared by diluting 100 mg/L of the individual a.i. stock solutions in 20 mL of ACN in a brown volumetric flask. The solution was stored at 4 °C protected from light (shelf life 30 days). Working solutions were prepared at concentrations ranging from 0.0001 to 0.1 mg/L by serial dilution (dilution factor × 10) in ACN.

Following the procedure described in Section 2.3, untreated blank samples were processed to obtain matrix extracts from both grain and straw. The absence of interference peaks from the target analytes in these extracts was confirmed by UHPLC-MS/MS analysis. According to the method, matrix-matched standard curves (0.0001 to 0.1 mg/L) were prepared using these matrix extracts as the dilution medium instead of ACN.

For method validation, blank grain and straw samples were separately spiked with the mixed standard solution at three concentration levels (0.002, 0.01, and 1.0 mg/kg). After mixing thoroughly, the samples were left to stand for 30 min to allow the solvent to evaporate, and then analyzed according to Section 2.3. Five replicate samples were processed at each concentration level within the same day for intra-day precision, while three replicates were analyzed on three consecutive days for inter-day precision. Untreated blank matrix samples were simultaneously processed as controls.

### 2.5. Storage Stability Test

To assess the storage stability of diflufenican, pyroxasulfone, and their metabolites (M-1, M-3, and M-25) in wheat grain and straw, the stability of these compounds was investigated according to the *Guideline for Storage Stability Testing of Pesticide Residues in Plant-Derived Agricultural Products* (NY/T 3094-2017) [44], using a fortified-sample storage experiment.

For grain samples at a fortification level of 0.5 mg/kg, five aliquots of 5.00 g blank matrix were accurately weighed into 50 mL PTFE centrifuge tubes, and each tube was individually spiked with 0.10 mL of a 25 mg/L standard solution corresponding to one of the analytes (diflufenican, pyroxasulfone, M-1, M-3, or M-25). After thorough homogenization, the tubes were sealed with parafilm and stored in a freezer at ≤−18 °C. For straw samples fortified at the same level (0.5 mg/kg), 2.0 g of blank matrix was weighed and spiked with 0.10 mL of a 10 mg/L mixed standard solution of the analytes, followed by identical mixing, sealing, and storage procedures.

Sampling was conducted at six designated time points: 0 days, 1 month, 2 months, 3 months, 6 months, and 12 months. At each interval, two fortified parallel samples and one blank control were analyzed, with an additional set of three backup samples (consisting of two fortified and one blank) prepared to account for potential experimental deviations. Throughout the storage duration, environmental conditions were rigorously controlled at ≤−18 °C and 30–50% relative humidity, with monthly monitoring of these parameters. All samples were analyzed within 24 h after retrieval.

In this study, the interference levels in all blank samples remained below 30% of the LOQ for the target analytes. This outcome confirms that matrix interference and background contamination were effectively controlled throughout the analytical process, meeting the methodological requirements for stability and ensuring the accuracy and reliability of the results.

### 2.6. Data Processing Methods

Residue quantification data were processed using the Mass Lynx V 4.2 system (Waters, USA), which is dedicated to the acquisition and analysis of UHPLC-MS/MS data. The matrix effect (ME%) was calculated according to established methods reported in the literature [45]. Statistical analysis and data visualization were performed using Microsoft Excel 2024 and Origin 2022, respectively.

The degradation rate *(DR, %)* of the stored samples was calculated using Equation (1):

*DR* = (*C*_0_ − *C_t_*)/*C*_0_ × 100% (1)

Here, the *DR* of the compound during storage is defined based on its concentration over time, where *C_t_* (mg/kg) is the concentration at time *t* (days) and *C*_0_ (mg/kg) is the initial spiked concentration (at *t* = 0). A *DR* of 30% was set as the stability threshold, with samples below this value considered stable and those above it deemed unstable.

The total residue (*C_T_*, mg/kg) of pyroxasulfone in plant-derived foods, is calculated as follows:

*C_T_* = *C_p_* + 1.261 × *C_M_*_-1_ + 1.504 × *C_M_*_-3_ + 1.321 × *C_M_*_-25_ (2)

In the formula, *C_p_*, *C_M-_*_1_, *C_M-_*_3_ and *C_M-_*_25_ denote the concentrations (mg/kg) of pyroxasulfone, M-1, M-3, and M-25, respectively; the coefficients 1.261, 1.504, and 1.321 are conversion factors derived from the molecular weight ratios of pyroxasulfone (391.30) to its metabolites (M-1: 310.20, M-3: 260.12, M-25: 296.17). These coefficients standardize all residue quantities as “pyroxasulfone equivalents”, serving as a metrological normalization rather than an indicator of toxicological potency.

The chronic dietary risk was assessed by calculating the risk quotient (*RQ*), defined as the ratio of the National Estimated Daily Intake (NEDI, mg/kg bw) to the Acceptable Daily Intake (ADI, mg/kg bw), expressed as a percentage [46]. NEDI was derived using Equation (3).

NEDI = ∑STMR × F_i_/bw (3)

The risk quotient (*RQ*) was calculated using Equation (4):

*RQ* = NEDI/ADI × 100% (4)

where STMR is a supervised trials median residue (mg/kg), ADI (acceptable daily intake) is the toxicological reference value (mg/kg bw), F_i_ represents the daily food consumption (kg/day) derived from the Chinese Resident Dietary Survey, with the corresponding data sourced from the *Report on Nutrition and Health Status of Chinese Residents* [47], and bw is the average body weight of an adult (63 kg). An *RQ* value of less than 100% indicates that the chronic dietary risk is acceptable. Conversely, an *RQ* value exceeding 100% suggests a potential health risk with unacceptable adverse effects on human health.

## 3. Results and Discussion

### 3.1. Linearity, Accuracy and Precision of the Method

The ionization modes for diflufenican, pyroxasulfone and its metabolites M-1, M-3, and M-25 were first optimized. In positive ion mode, diflufenican and pyroxasulfone exhibited efficient ionization to form prominent [M+H]^+^ ions, whereas metabolites M-1, M-2, and M-3 showed lower responses, likely due to the scarcity of readily protonatable sites within their molecular structures. In negative ion mode, metabolites M-1, M-3, and M-25 displayed significantly enhanced signals attributed to the presence of easily deprotonated functional groups, while the response of diflufenican and pyroxasulfone declined considerably. The inclusion of 0.1% formic acid in the mobile phase substantially improved detection sensitivity in both modes of ionization. This enhancement is ascribed to the dual function of formic acid: serving as a proton donor to promote positive ionization, and forming formate adduct ions [M+HCOO]^−^ to strengthen detection in negative ion mode. Following precursor ion optimization, the Intellistart automated procedure was employed for product ion scanning. Through ion abundance screening, the two most sensitive ion pairs (quantitative and qualitative) were selected, while key mass spectrometric parameters, including cone voltage and collision energy, were simultaneously optimized via the instrument’s automated module.

Subsequently, LC conditions were optimized after establishing mass spectrometric parameters for diflufenican, pyroxasulfone, and their metabolites. Experimental results demonstrated that the methanol–water system, owing to its high viscosity and weak eluting strength, not only reduced the responses of the five target analytes but also significantly elevated system backpressure. In contrast, the acetonitrile–water system provided superior separation performance. The addition of 0.1% formic acid to the acetonitrile—water system markedly enhanced the sensitivity of all five compounds, primarily due to formic acid’s ability to adjust mobile–phase pH to favor protonation while suppressing secondary interactions with residual silanol groups on the stationary phase, thereby improving peak shapes. To address macromolecular interferences such as polysaccharides and pigments in wheat samples, a reversed-phase gradient elution program was implemented. The initial 95% aqueous phase (0.1% formic acid) rapidly removed polar interferences, followed by a linear increase in acetonitrile proportion over 3 min. This approach successfully confined the retention times of the target analytes within an ideal window of 2.0–4.0 min. Once these conditions were verified to satisfy the research requirements, no further optimization was pursued.

Under the finalized instrumental setup, all target analytes displayed excellent linearity across a concentration range of 0.0001–0.1 mg/L, with correlation coefficients (*r*) exceeding 0.996. As shown in Table 4, significant matrix effects were observed for both diflufenican and pyroxasulfone in the wheat grain matrix. Compared with the acetonitrile solvent system, the slope for diflufenican decreased by 47.5% in the grain matrix, while the matrix effect in the straw matrix was negligible (approximately 0.8%). Similarly, the slopes for pyroxasulfone decreased by 44.6% in the grain matrix and 29.0% in the straw matrix. The three metabolites exhibited distinct matrix effect patterns. Metabolite M-25, which showed the highest slope in the acetonitrile solvent system, decreased by only 9.3% in the grain matrix and 1.7% in the straw matrix. These values meet the acceptable criterion of |ME| < 10%, indicating negligible matrix interference for this metabolite. In contrast, metabolites M-1 and M-3 demonstrated matrix enhancement effects. Compared with the solvent system, the slopes of M-1 increased by 16.2% in the grain matrix and 26.7% in the straw matrix, while those of M-3 increased by 14.5% and 14.3%, respectively.

The observed matrix suppression or enhancement is likely attributable to interference from lipids, phenolic compounds, or other specific components present in the wheat grain and straw matrices, which may influence electrospray ionization efficiency through mechanisms such as charge transfer or competitive ionization. Due to the substantial variation in response characteristics under different matrix conditions, a matrix-matched external standard method was employed for quantification in this study to correct for matrix effects and ensure the accuracy and reliability of the analytical results.

Method validation was conducted by evaluating accuracy (recovery) and precision at three fortification levels (0.002, 0.01, and 0.5 mg/kg) in both wheat grain and straw matrices. Mean recoveries for diflufenican, pyroxasulfone, M-1, M-3, and M-25 were 81.6–104.1%, 82.0–104.3%, 95.3–106.9%, 99.1–104.4%, and 95.6–110.1%, respectively. Intra-day precision (RSD_a_, *n* = 5) ranged from 0.9% to 4.4%, while inter-day precision (RSD_b_, *n* = 9) ranged from 1.3% to 13.9%. All validation parameters met the acceptance criteria (recovery: 70–120%; RSD ≤ 20%) established in Chinese and European guidelines [43,48].

For samples exceeding the upper limit of the linear calibration range, a validated 10-fold dilution approach was applied, and the resulting recoveries consistently remained within acceptable levels. Due to the influence of matrix effects, the validated LOQ for this method was established at 0.002 mg/kg. Although further optimization might enable a lower method detection limit, the current LOQ meets the analytical requirements of this study, and thus no further investigation was undertaken in this study.

**Figure 2:**
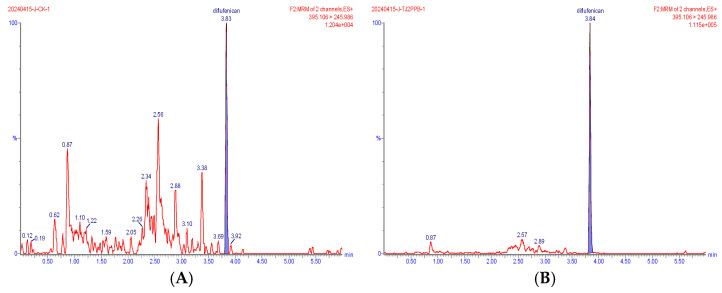
MRM chromatograms of diflufenican in blank matrix (**A**) and spiked sample at 0.002 mg/kg (**B**).

**Figure 3:**
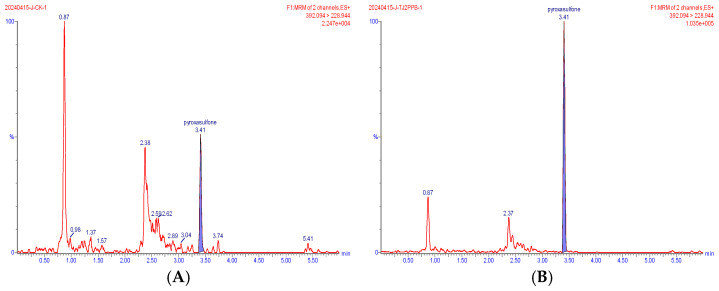
MRM chromatograms of pyroxasulfone in blank matrix (**A**) and spiked sample at 0.002 mg/kg (**B**).

**Figure 4:**
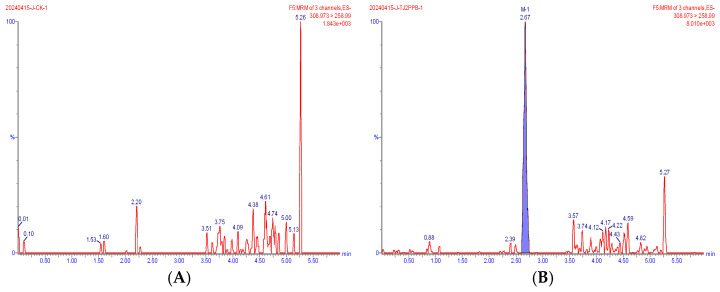
MRM chromatograms of M-1 in blank matrix (**A**) and spiked sample at 0.002 mg/kg (**B**).

**Figure 5:**
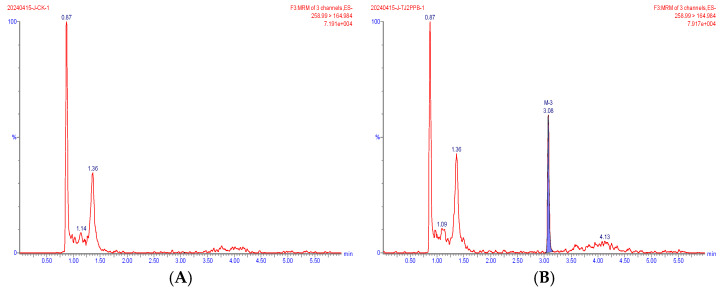
MRM chromatograms of M-3 in blank matrix (**A**) and spiked sample at 0.002 mg/kg (**B**).

**Figure 6:**
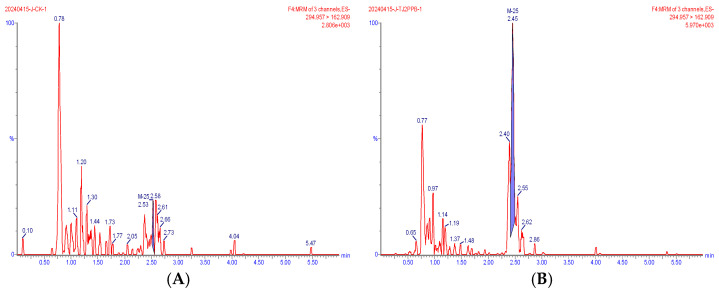
MRM chromatograms of M-25 in blank matrix (**A**) and spiked sample at 0.002 mg/kg (**B**).

Representative extracted ion chromatograms of the quantifier transitions for the five compounds detected in wheat are shown in Figure 2, Figure 3, Figure 4, Figure 5 and Figure 6. In the MRM chromatogram, the target compound appears as a blue peak, while the interfering substances/impurities are visible as red peaks.

### 3.2. Assessment of the Storage Stability of the Herbicide

A systematic evaluation of the storage stability of diflufenican, pyroxasulfone, and their metabolites (M-1, M-3, and M-25) in wheat grain and straw was conducted. Samples were fortified at 0.5 mg/kg and stored for 12 months at ≤−18 °C. The results showed that the recoveries of all target analytes in quality control samples met the established criteria for pesticide residue analysis, as summarized in Table 5.

In grain, the *DR* of all compounds remained below the 30% stability threshold. Pyroxasulfone exhibited the highest *DR* (27%) after 3 months of storage. A similar trend was observed in straw, with the *DR* of all compounds remaining below 30%. Notably, the DR of pyroxasulfone in straw reached 29% after 12 months of storage, approaching the upper limit of the stability threshold. In addition, minor negative degradation values (−8% to −1%) observed for metabolites M-1 and M-3 at certain time points fell within the acceptable variation range confirmed during method validation. In conclusion, the results confirm the stability of the target compounds in wheat grain and straw over 12 months of storage and demonstrate that the residue data accurately reflect their initial concentrations throughout the test period.

### 3.3. Terminal Residues of the Herbicides

The final residue concentrations of diflufenican, pyroxasulfone, and their metabolites (M-1, M-3, and M-25) in wheat straw and grain from 10 experimental sites across the country are summarized in Table 6.

In wheat grain, residues of diflufenican were all below the LOQ (0.002 mg/kg), and the STMR was determined as <0.002 mg/kg. In contrast, residue concentrations in straw were generally higher and exhibited greater variability, with measured values ranging from 0.002 to 0.018 mg/kg (STMR: 0.010 mg/kg). The highest residue level (0.018 mg/kg) was observed in samples from Yunnan province. GB 2763 [23] stipulates a MRL of 0.05 mg/kg for diflufenican in wheat. The results of this study demonstrate that residue levels of diflufenican in both wheat grain and straw consistently remained below this regulatory limit, thereby confirming that diflufenican residues in wheat comply with current national food safety standards.

In wheat grain, the concentration of pyroxasulfone ranged from <0.002 to 0.010 mg/kg, corresponding to an STMR of <0.002 mg/kg. Its metabolite M-1 was consistently detected at a concentration of 0.002 mg/kg across all trial sites, corresponding to an STMR of 0.002 mg/kg. Concentrations of metabolites M-3 and M-25 ranged from 0.002 to 0.006 mg/kg and 0.002 to 0.005 mg/kg, respectively, each with an STMR of 0.002 mg/kg. In straw, the residue concentrations of pyroxasulfone ranged from <0.002 to 0.010 mg/kg (STMR: <0.002 mg/kg), with the highest value (0.010 mg/kg) also observed in Yunnan. The concentration of metabolite M-1 in straw varied from 0.005 to 0.221 mg/kg (STMR: 0.044 mg/kg), and the maximum concentration (0.221 mg/kg) was detected in Yantai, Shandong. Concentrations of M-3 and M-25 in straw ranged from 0.005 to 0.019 mg/kg (STMR: 0.009 mg/kg) and from 0.005 to 0.048 mg/kg (STMR: 0.017 mg/kg), respectively, with their peak values (0.019 mg/kg for M-3 and 0.048 mg/kg for M-25) both recorded in Yunnan.

Currently, the Joint FAO/WHO Meeting on Pesticide Residues (JMPR) [49] has not yet established assessment criteria for pyroxasulfone. Major regulatory bodies, including China, the CAC, and the EU, have not set MRLs for pyroxasulfone in wheat. In accordance with the established international MRLs, the residue concentration of pyroxasulfone quantified in this trial were found to comply with the standards set by the EPA, Japan, and Republic of Korea. However, these levels exceeded the MRL established for pyroxasulfone in Australia.

### 3.4. Dietary Risk Assessment

To date, no studies or regulatory reports have indicated that the toxicity of metabolites M-1, M-3, or M-25 exceeds that of the parent compound pyroxasulfone. Given that these metabolites share the same active functional groups and core pharmacophore structure with the parent compound, and in the absence of comprehensive toxicological data for individual metabolites, this study adopted a conservative risk assessment strategy by defining the total residue of pyroxasulfone as the sum of the parent compound and its metabolites M-1, M-3, and M-25. According to this definition, residue results from ten trial sites showed that the total pyroxasulfone residue in wheat grain ranged from 0.010 to 0.019 mg/kg (STMR: 0.014 mg/kg) and in straw from 0.026 to 0.357 mg/kg (STMR: 0.092 mg/kg).

GB 2763 [23] stipulates an ADI of 0.2 mg/kg bw for diflufenican but does not provide an ADI for pyroxasulfone. Based on the only publicly available official data from the U.S. EPA [26], the ADI for pyroxasulfone is 0.02 mg/kg bw. Based on the current status of pesticide registration in China and the average dietary structure model of the population, the calculated NEDI for diflufenican in the general population is 1.962 × 10^−4^ mg/kg bw, with a RQ of 0.10%; for pyroxasulfone, the NEDI is 5.341 × 10^−5^ mg/kg bw, with an RQ of 0.27% (Table 7). The dietary intake risk assessment results for both compounds are far below 100%, indicating that the application of 200 g/L pyroxasulfone–diflufenican SC in accordance with GAP does not pose an unacceptable risk to the health of the general population.

Currently, China has not established MRL standards for pyroxasulfone. Based on the assessment results and the final residue data of this study, it is recommended that the residue definition for pyroxasulfone be defined as the sum of the parent compound and its metabolites M-1, M-3, and M-25. Furthermore, it is suggested that the MRL for pyroxasulfone be set at 0.03 mg/kg in wheat grain and at 0.6 mg/kg in wheat straw. These recommended values are consistent with international standards while also meeting the practical needs of agricultural production and regulatory oversight in China.

## 4. Conclusions

A reliable analytical method was developed using QuEChERS sample preparation coupled with UHPLC-MS/MS, achieving a LOQ of 0.002 mg/kg. This method enables the simultaneous determination of diflufenican, pyroxasulfone, and their metabolites (M-1, M-3, and M-25) in wheat grain and straw. Method validation, conducted on spiked samples under controlled laboratory conditions, demonstrated satisfactory accuracy and precision. All target analytes remained stable in wheat matrices during storage at −18 °C for 12 months, ensuring the robustness of subsequent residue data.

Supervised field trials were performed during the 2023–2024 winter wheat growing season across ten representative agricultural regions in China. A single pre-emergence soil application of 200 g/L pyroxasulfone–diflufenican SC was carried out at the maximum label dose of 480 g a.i./ha. At harvest, residues of diflufenican in wheat grain were all below the LOQ (<0.002 mg/kg), corresponding to a supervised trials median residue (STMR) of <0.002 mg/kg—substantially lower than the current Chinese MRL of 0.05 mg/kg. Residues in straw ranged from 0.002 to 0.018 mg/kg (STMR 0.010 mg/kg), also well below the established regulatory limit. For pyroxasulfone, for which no MRL has yet been established in China, it is recommended that the residue definition be set as the sum of the parent compound and its metabolites M-1, M-3, and M-25. Based on this definition, total residues in wheat grain ranged from 0.010 to 0.019 mg/kg (STMR 0.014 mg/kg), and in straw from 0.026 to 0.357 mg/kg (STMR 0.092 mg/kg). Dietary intake risk assessment, based on the registered use pattern and the average dietary structure of the Chinese population, revealed NEDI of 1.962 × 10^−4^ mg/kg bw for diflufenican and 5.341 × 10^−5^ mg/kg bw for pyroxasulfone, with corresponding RQ of 0.10% and 0.27%, respectively. Both values are well below 100%, indicating that the application of the formulation in accordance with good agricultural practices (GAP) poses no unacceptable health risk to consumers.

Based on the residue trial data and dietary risk assessment, it is recommended that China establish MRLs for pyroxasulfone in wheat at 0.03 mg/kg for grain and 0.6 mg/kg for straw. These proposed limits are consistent with international standards and address both domestic agricultural practices and regulatory requirements, providing a scientific basis for future MRL setting and revision.

## Figures and Tables

**Table 1:** Comparison of MRLs for pyroxasulfone and diflufenican in crops among international standards.

Compound	Crop	MRLs (mg/kg)						
		China [23,24]	CAC [25]	EPA [26]	Australia [27]	Republic of Korea [28]	EU [29,30]	Japan [31]
Pyroxasulfone	Wheat	/	/	0.03	0.01	/	/	0.01
	Corn	/	/	0.02	0.01	/	/	0.01
	Soybeans	/	/	0.06	0.06	/	/	0.01
Diflufenican	Wheat	0.05	/	/	0.02	/	0.02	0.10
	Rice	0.05	/	/	0.20	/	0.01	0.05
	Garlic	/	/	/	/	/	0.01	/

CAC: Codex Alimentarius Commission; EPA: U.S. Environmental Protection Agency; EU: European Union.

**Table 2:** Field trial parameters for winter wheat experiments.

Location	Wheat Variety	Soil Type	pH	OA (g/kg)	CEC (cmol/kg)	Spraying Date	Sampling Date	Analysis Date
Shanxi	Yingbo 700	Loam	8.3	17.2	15.3	9 October 2023	25 June 2024	22 August 2024
Gansu	Longzhong No. 6	Loam	6.8	6.7	14.7	28 September 2023	8 July 2024	22 August 2024
Beijing	Jima 22	Loam	7.9	18.2	14.5	10 October 2023	18 June 2024	22 August 2024
Henan	Zhongmai 895	Fluvo-aquic soil	7.2	23.9	12.5	2 November 2023	5 June 2024	22 August 2024
SD Weifang	Jimai 21	Loam	8.0	12.7	17.7	2 October 2023	30 May 2024	21 August 2024
SD Yantai	Yannong 999	Brown soil	7.5	15.3	16.4	16 October 2023	31 May 2024	21 August 2024
Anhui	Zhenmai 18	Clayey fluvo-aquic soil	7.1	15.0	16.3	31 October 2023	29 May 2024	21 August 2024
Shanghai	Yangmai 26	Fluvo-aquic soi	7.8	30.1	19.4	23 November 2023	3 June 2024	22 August 2024
Hunan	Xinong 511	Red soil	5.3	31.2	13.0	31 October 2023	13 May 2024	21 August 2024
Yunnan	Yunmai 77	Loam	7.6	24.7	11.9	14 November 2023	2 May 2024	21 August 2024

OA: organic matter content; CEC: cation exchange capacity; SD: Shangdong.

**Table 3:** UHPLC-MS/MS parameters for diflufenican, pyroxasulfone, M-1, M-3, and M-25.

Compound	Molecular Formula	Retention Time	Qualitative Ion Pairs (*m*/*z*)	Quantitative Ion Pairs (*m*/*z*)	Cone Voltage (V)	Collision Energy (V)
Diflufenican	C_19_H_11_F_5_N_2_O_2_	3.83	395.1/246.0	395.1/246.0	16	40
			395.1/265.9		16	24
Pyroxasulfone	C_12_H_14_F_5_N_3_O_4_S	3.41	392.1/179.0	392.1/228.9	36	28
			392.1/228.9		36	16
M-1	C_7_H_7_F_5_N_2_O_4_S	2.45	309.0/195.2	309.0/259.0	6	18
			309.0/259.0		6	17
M-3	C_7_H_5_F_5_N_2_O_3_	3.07	259.0/149.9	259.0/165.0	2	38
			259.0/165.0		2	16
M-25	C_6_H_5_F_5_N_2_O_4_S	2.44	295.0/162.9	295.0/162.9	8	20
			295.0/231.0		8	16

**Table 4:** Analytical parameters for diflufenican, pyroxasulfone, M-1, M-3, and M-25 in grain and straw.

Compound	Linear Equation (Matrix)	ME	Fortification	Mean Recovery in Grain (%)	Mean Recovery in Wheat (%)	LOQ
	(y = ax + b)	(%)	Level (mg/kg)			(mg/kg)
Diflufenican	y = 9826.2x − 2045 (ACN)	/	0.002	104.1	100.3	/
	y = 5158.7x + 479.84 (Grain)	−47.5	0.01	93.9	95.4	0.002
	y = 9903.1x + 2669.4 (Straw)	0.8	0.5	88.5	81.6	0.002
Pyroxasulfone	y = 10980x − 3219.6 (ACN)	/	0.002	102.6	104.3	/
	y = 6087.8x − 5481.1 (Grain)	−44.6	0.01	100.8	98.4	0.002
	y = 7794.4x − 4482.4 (Straw)	−29	0.5	82.0	92.0	0.002
M-1	y = 694.1x + 35.028 (ACN)	/	0.002	95.5	95.3	/
	y = 806.42x + 162.44 (Grain)	16.2	0.01	97.3	100.4	0.002
	y = 879.68x + 53.705 (Straw)	26.7	0.5	106.9	99.6	0.002
M-3	y = 2138.1x + 768.29 (ACN)	/	0.002	102.1	101.6	/
	y = 2447.3x − 83.809 (Grain)	14.5	0.01	101.5	99.1	0.002
	y = 2443x + 1103.3 (Straw)	14.3	0.5	104.4	103.2	0.002
M-25	y = 1080.3x + 110.51 (ACN)	/	0.002	110.1	103.7	/
	y = 979.34x − 266.93 (Grain)	−9.3	0.01	98.7	95.7	0.002
	y = 1062.2x − 25.326 (Straw)	−1.7	0.5	95.6	101.2	0.002

**Table 5:** Stability of the five target compounds in wheat grain and straw matrices under cold storage conditions.

Compound	Storage Time	Wheat Grain			Wheat Straw		
	(Months)	Concentration	*DR*	QC Recovery	Concentration	*DR*	QC Recovery
		(mg/kg)	(%)	(%)	(mg/kg)	(%)	(%)
Diflufenican	0	0.513 ± 0.051	−3	98	0.443 ± 0.016	11	102
	1	0.421 ± 0.062	16	99	0.526 ± 0.013	−5	87
	3	0.414 ± 0.023	17	97	0.511 ± 0.021	−2	94
	6	0.453 ± 0.036	10	84	0.491 ± 0.021	2	85
	12	0.471 ± 0.024	6	84	0.402 ± 0.008	20	74
Pyroxasulfone	0	0.474 ± 0.004	5	93	0.443 ± 0.016	11	93
	1	0.387 ± 0.007	23	82	0.450 ± 0.033	10	82
	3	0.365 ± 0.004	27	85	0.488 ± 0.048	2	85
	6	0.422 ± 0.067	16	95	0.451 ± 0.016	10	95
	12	0.428 ± 0.019	14	93	0.356 ± 0.003	29	93
M-1	0	0.519 ± 0.033	−4	91	0.538 ± 0.016	−8	91
	1	0.530 ± 0.008	−6	94	0.451 ± 0.010	10	94
	3	0.503 ± 0.016	−1	103	0.437 ± 0.016	13	103
	6	0.397 ± 0.016	21	85	0.506 ± 0.045	−1	85
	12	0.423 ± 0.025	15	90	0.430 ± 0.017	14	90
M-3	0	0.504 ± 0.011	−1	93	0.470 ± 0.002	6	93
	1	0.528 ± 0.015	−6	104	0.450 ± 0.008	10	104
	3	0.516 ± 0.013	−3	99	0.423 ± 0.004	15	99
	6	0.442 ± 0.052	12	82	0.505 ± 0.018	−1	82
	12	0.485 ± 0.027	3	81	0.524 ± 0.006	−5	81
M-25	0	0.462 ± 0.028	8	95	0.426 ± 0.004	15	95
	1	0.430 ± 0.002	14	95	0.419 ± 0.037	16	95
	3	0.411 ± 0.025	18	95	0.450 ± 0.014	10	95
	6	0.477 ± 0.047	5	79	0.365 ± 0.018	27	79
	12	0.387 ± 0.031	23	77	0.449 ± 0.030	10	77

**Table 6:** Final residues of diflufenican, pyroxasulfone and their metabolites in wheat grain and straw.

Location	Dosage (g a.i./ha)	Spray Times	Grain Concentration (mg/kg)					Straw Concentration (mg/kg)				
			Diflufenican	Pyroxasulfone	M-1	M-3	M-25	Diflufenican	Pyroxasulfone	M-1	M-3	M-25
Shanxi	480	1	<LOQ	0.010	0.002	0.002	<LOQ	0.003	<LOQ	0.008	0.005	0.005
Gansu	480	1	<LOQ	<LOQ	0.002	<LOQ	0.005	0.003	<LOQ	0.009	0.005	0.005
Beijing	480	1	<LOQ	<LOQ	0.002	0.005	<LOQ	0.010	<LOQ	0.084	0.012	0.019
Henan	480	1	<LOQ	<LOQ	0.002	0.002	0.002	0.010	<LOQ	0.037	0.011	0.016
SD Weifang	480	1	<LOQ	0.006	0.002	0.002	<LOQ	0.017	<LOQ	0.050	0.006	0.017
SD Yantai	480	1	<LOQ	0.007	0.002	0.004	0.003	0.015	0.004	0.221	0.010	0.045
Anhui	480	1	<LOQ	<LOQ	0.002	0.006	0.002	0.014	<LOQ	0.144	0.016	0.031
Shanghai	480	1	<LOQ	<LOQ	0.002	<LOQ	<LOQ	0.004	<LOQ	0.005	0.007	0.006
Hunan	480	1	<LOQ	<LOQ	0.002	<LOQ	<LOQ	0.002	<LOQ	0.005	0.008	0.006
Yunnan	480	1	<LOQ	<LOQ	0.002	0.002	<LOQ	0.018	0.010	0.095	0.019	0.048

**Table 7:** The dietary intake risk assessment of diflufenican and pyroxasulfone.

Food Classification	Fi (kg/Day)	Diflufenican				Pyroxasulfone			
		Reference Limits (mg/kg)	Sources	NEDI (mg/kg bw)	RQ (%)	Reference Limits (mg/kg)	Sources	NEDI (mg/kg bw)	RQ (%)
Rice and Its Products	0.2399	0.05	China	1.904 × 10^−4^	0.10	/	/	/	0.27
Wheat and Its Products	0.1385	0.002	STMR	4.397 × 10^−6^		0.014	STMR	3.078 × 10^−5^	
Other Cereals	0.0233	/	/	/		0.02	USA	7.397 × 10^−6^	
Potatoes and Tubers	0.0495	/	/	/		/	/	/	
Legumes and Their Products	0.0160	/	/	/		0.06	USA	1.524 × 10^−5^	
Dark-colored Vegetables	0.0915	/	/	/		/	/	/	
Light-colored Vegetables	0.1837	/	/	/		/	/	/	
Pickled Vegetables	0.0103	/	/	/		/	/	/	
Fruits	0.0457	/	/	/		/	/	/	
Nuts	0.0039	/	/	/		/	/	/	
Vegetable Oil	0.0327	/	/	/		/	/	/	
Salt	0.0120	/	/	/		/	/	/	
Soy Sauce	0.0090	0.01	EU	1.429 × 10^−6^		/	/	/	
Total				1.962 × 10^−4^				5.341 × 10^−5^	

## Data Availability

The original contributions presented in the study are included in the article; further inquiries can be directed to the corresponding authors.

## Abbreviations

The following abbreviations are used in this manuscript:

UHPLC-MS/MS	Ultra-high-performance liquid chromatography–tandem mass spectrometry
QuEChERS	Quick, Easy, Cheap, Effective, Rugged, Safe
MRM	Multiple Reaction Monitoring
NEDI	National Estimated Daily Intake
MRL	Maximum Residue Limit
GAP	Good Agricultural Practice
CAC	Codex Alimentarius Commission
LOQ	Limit of Quantification
EPA	Environmental Protection Agency
